# The influence of genetic variation in 30 selected genes on the clinical characteristics of early onset breast cancer

**DOI:** 10.1186/bcr2213

**Published:** 2008-12-18

**Authors:** William Tapper, Victoria Hammond, Sue Gerty, Sarah Ennis, Peter Simmonds, Andrew Collins, the Prospective study of Outcomes in Sporadic versus Hereditary breast cancer (POSH) Steering Group, Diana Eccles

**Affiliations:** 1 Human Genetics and Cancer Sciences Divisions, School of Medicine, Southampton General Hospital, University of Southampton, Southampton, SO16 6YD, UK

## Abstract

**Introduction:**

Common variants that alter breast cancer risk are being discovered. Here, we determine how these variants influence breast cancer prognosis, risk and tumour characteristics.

**Methods:**

We selected 1,001 women with early onset nonfamilial invasive breast cancer from the Prospective study of Outcomes in Sporadic versus Hereditary breast cancer (POSH) cohort and genotyped 206 single nucleotide polymorphisms (SNPs) across 30 candidate genes. After quality control, 899 cases and 133 SNPs remained. Survival analyses were used to identify SNPs associated with prognosis and determine their interdependency with recognized prognostic factors. To identify SNPs that alter breast cancer risk, association tests were used to compare cases with controls from the Wellcome Trust Case Control Consortium. To search for SNPs affecting tumour biology, cases were stratified into subgroups according to oestrogen receptor (ER) status and grade and tested for association.

**Results:**

We confirmed previous associations between increased breast cancer risk and SNPs in *CASP8*, *TOX3 *(previously known as *TNRC9*) and *ESR1*. Analysis of prognosis identified eight SNPs in six genes (*MAP3K1*, *DAPK1*, *LSP1*, *MMP7*, *TOX3 *and *ESR1*) and one region without genes on 8q24 that are associated with survival. For *MMP7*, *TOX3 *and *MAP3K1 *the effects on survival are independent of the main recognized clinical prognostic factors. The SNP in 8q24 is more weakly associated with independent effects on survival. Once grade and pathological nodal status (pN stage) were taken into account, SNPs in *ESR1 *and *LSP1 *showed no independent survival difference, whereas the effects of the *DAPK1 *SNP were removed when correcting for ER status. Interestingly, effects on survival for SNPs in *ESR1 *were most significant when only ER-positive tumours were examined. Stratifying POSH cases by tumour characteristics identified SNPs in *FGFR2 *and *TOX3 *associated with ER-positive disease and SNPs in *ATM *associated with ER-negative disease.

**Conclusions:**

We have demonstrated that several SNPs are associated with survival. In some cases this appears to be due to an effect on tumour characteristics known to have a bearing on prognosis; in other cases the effect appears to be independent of these prognostic factors. These findings require validatation by further studies in similar patient groups.

## Introduction

Breast cancer arises as a result of multiple somatic molecular events that can be genetic or epigenetic. Further research is required to define the inherited factors that contribute to breast cancer risk. To date, six genes associated with high risk (*BRCA1*, *BRCA2*, *TP53*, *PTEN*, *STK11 *and *CDH1*), four associated with modest risk (*PALB2*, *BRIP1*, *ATM *and *CHEK2*) and six lower penetrance alleles (*CASP8*, *FGFR2*, *TOX3*, *MAP3K1*, *LSP1 *and 8q24 rs13281615) have been identified using various approaches [1-5]. Genetic variability appears to influence not only risk but also the type of breast cancer that develops in an individual. There is compelling data that pathogenic mutations in *BRCA1 *result in a distinct tumour phenotype, whereas more subtle similarities are seen between *BRCA2 *cases and among familial non-*BRCA1*/*BRCA2 *cancers [6-9]. In studies of lower penetrance alleles it is clear that most of the increase in breast cancer risk is for oestrogen receptor (ER)-positive breast cancers (which form the majority of most breast cancer cohorts) [10-12]. There is some evidence that breast cancer prognosis may be influenced by inherited genetic factors. Ethnicity appears to be associated with tumour biology and outcome [13,14]. Recent data from a large Swedish population-based study indicated that the prognosis of mothers with breast cancer influenced the likelihood of survival in their daughters who subsequently developed breast cancer, suggesting an inherited component to prognosis [15]. It is also apparent that inherited genetic factors can influence drug metabolism, and this may affect prognosis after breast cancer diagnosis by influencing the efficacy of treatment [16,17].

As an example of this it is apparent that ER-modulating drugs reduce the risk for developing ER-positive breast cancer by up to 50%, but they do not alter the incidence of ER-negative breast cancer [18]. If a given genetic risk profile indicates a raised likelihood of developing ER-positive breast cancer specifically, then targeting this group of individuals with tamoxifen or raloxifene treatment as a prevention strategy would give the most benefit.

Early onset breast cancers are more likely to have arisen due to an inherited predisposition and tend to have a worse prognosis, possibly as a result of a different pattern of genomic expression compared with tumours developing in older women [19]. Women with early onset breast cancer are thus an ideal population in which to search for common genetic variants that may influence breast cancer risk and prognosis. The discovery of such genetic markers may allow clinicians to advise patients more accurately about appropriate prevention and screening strategies, tumour prognosis and treatment.

## Materials and methods

### Patient cohort

To identify SNPs associated with prognosis, risk and tumour characteristics, we selected 1,001 women from the Prospective study of Outcomes in Sporadic versus Hereditary breast cancer (POSH) cohort for genotyping of 30 genes with prior evidence for biological relevance [see Additional data file 1]. Full details of the POSH study can be obtained from the published protocol [20]. The subset of 1,001 POSH patients are all incident cases who were aged 40 years or younger at diagnosis of invasive carcinoma between January 2000 and December 2007 and have no family history of breast cancer or ovarian cancer (all recruits had submitted family history details, collected by questionnaire). These cases are therefore least likely to carry a high risk susceptibility allele such as *BRCA1 *or *BRCA2*. To date, comprehensive *BRCA1 *and *BRCA2 *mutation screening (using a combination of conformation sensitive capillary electrophoresis and multiplex ligation-dependent probe amplification) has been carried out in 120 patients from the POSH cohort, 39 of which reported no family history of breast or ovarian cancer. For those without family history, one in 39 (2.5%) had a pathogenic *BRCA1 *mutation and no pathogenic *BRCA2 *mutations were found (unpublished data).

This subset of POSH cases all presented symptomatically, which means the tumour biology and clinical features have not been modified by earlier detection in a screening programme. The effect of tumour biology on prognosis is reflected in the known clinical prognostic factors (tumour size and grade, ER status and pathological nodal [pN] status). These parameters are used to make decisions about adjuvant therapy [21] and have been recorded for all POSH cases. On the basis of these factors, the subset of POSH cases were stratified into subgroups and compared to identify common variants that affect tumour biology.

### Genotyping and quality control

A total of 206 tag single nucleotide polymorphisms (SNPs) across 30 genes were selected for genotyping, ensuring coverage of each gene and its promoter region with *r*^2 ^≥ 0.9 (*r*^2 ^between adjacent SNPs in candidate genes) using data from the HapMap project [22]. Genotyping was carried out by Sequenom (San Diego, CA, USA) using the iPLEX service, which is based on mass spectrometry. A little more than one-third of these SNPs (35%, 73) were removed by a screening procedure that rejected SNPs with ≥10% missing genotypes (60) or allele frequencies less than 5% (12). Typically, deviations from Hardy-Weinberg equilibrium in controls would be used to exclude SNPs. However, because only cases were genotyped, Hardy-Weinberg deviations may reflect association with breast cancer, rather than poor genotyping. We therefore verified genotyping calls by examining the Sequenom cluster plots for SNPs with significant deviations from Hardy-Weinberg equilibrium (χ^2 ^≥ 10). On this basis, one SNP was removed leaving 133 (64%) for analysis, which is consistent with the genotyping tier [see Additional data file 1]. A high proportion of missing genotypes per individual DNA sample may suggest relatively poor DNA quality. Most samples had very low rates of missing data, and we removed 58 individuals with ≥ 10% missing genotypes, leaving 943 for further analysis.

**Figure 1:**
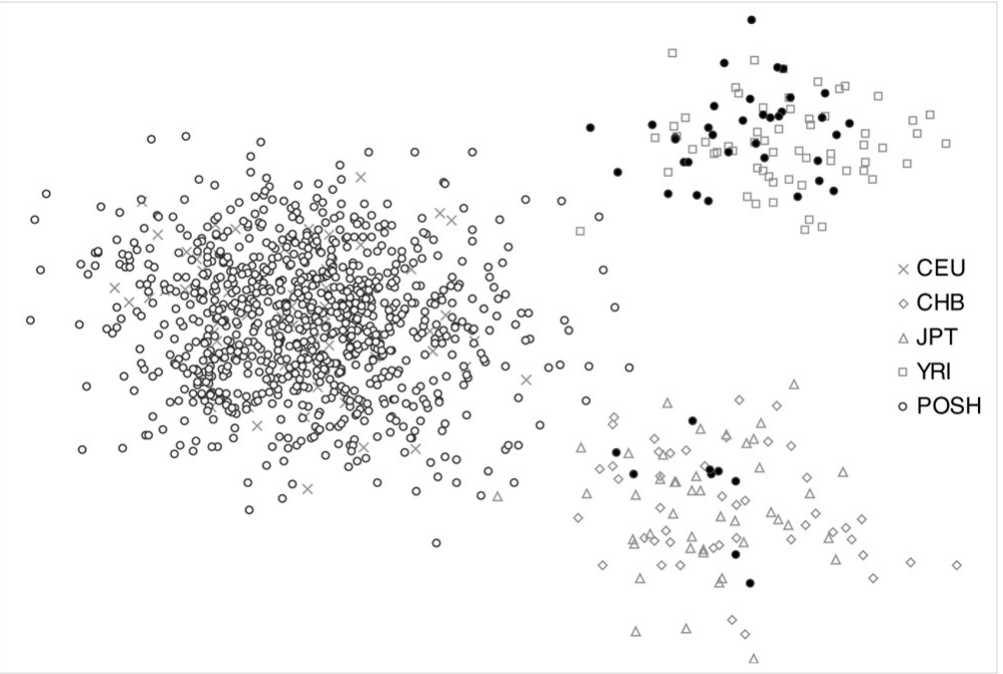
**Inference of ancestry by multidimensional scaling**. POSH and HapMap samples plotted for the first two principal components obtained by multidimensional scaling of a matrix of pairwise identity by state values. Excluded POSH samples, filled black circles, occur near the YRI and CHB+JPT clusters. CHB, Chinese; CUE, Western European; JPT, Japanese; POSH, Prospective study of Outcomes in Sporadic versus Hereditary breast cancer; YRI, Nigerian.

Because population stratification may cause false positives in association studies, and the majority of individuals in the POSH cohort have Western European ancestries, we removed individuals with apparently different ancestries. We used the PLINK program [23] to perform multidimensional scaling on genome-wide average identities by state. To avoid confounding of the multidimensional scaling by including any nonindependent SNPs due to extended linkage disequilibrium, we thinned the data to 82 autosomal SNPs with maximum pair-wise *r*^2 ^≤ 0.2. These data were merged with data from the founders or unrelated individuals in the HapMap sample [22]: 60 Western European (CEU), 60 Nigerian (YRI), 45 Han Chinese (CHB) and 45 Japanese (JPT). Before merging the genotypic data from POSH and HapMap, we ensured that all genotypes were configured to the positive strand of the reference sequence (National Cancer for Biotechnology Information [NCBI] build 36.1). Although more than 10,000 autosomal SNPs are recommended for this type of analysis [23], plotting the first two components from the multidimensional scaling analysis of 82 SNPs, which represent geographic and genetic variation, clearly identifies three distinct clusters that correspond to African, Asian, and Western European ancestries (Figure 1). Given this, we were able to exclude 44 samples (35 African and 9 Asian) that did not align with the Western European cluster, leaving 899 for analysis. The genotyping call rate for the 133 SNPs and 899 individuals passing our quality control procedure is 99%.

### Statistical analysis

To identify SNPs influencing prognosis and because the median follow-up time for the 899 patients passing quality control is only 2.4 years (Table 1), we calculated distant disease-free survival (DDFS) times that we define as the time between diagnosis and the first distant metastasis. This is a surrogate for overall survival because the majority of patients with distant metastases will not survive the disease. In seven cases, the date of death was used because the date of distant metastasis was not available. In one of these cases the cause of death was known to be unrelated to breast cancer. Univariate analyses of DDFS were performed using the log rank test to compare Kaplan-Meier DDFS curves for the three genotypes of each SNP.

**Table 1:** Distribution of prognostic phenotypes in 899 patients from the POSH cohort according to outcome

Outcome	Number	DDFS^a^	ER status^b^		Tumour grade^b^			Diameter (cM)	pN stage^b^	
						
			Negative	Positive	1	2	3		1	0
Deceased	102	1.48 (0.00 to 4.77)	53 (53.0)	47 (47.0)	2 (2.0)	19 (19.2)	78 (78.8)	3.67	70 (76.9)	21 (23.1)
Distant relapse	55	1.90 (0.00 to 4.62)	18 (32.7)	37 (67.3)	2 (3.8)	18 (34.0)	33 (62.0)	3.00	38 (74.5)	13 (25.5)
Disease free	742	2.61 (0.05 to 6.69)	202 (27.6)	531 (72.4)	60 (8.2)	269 (36.8)	402 (55.0)	2.58	352 (48.4)	376 (51.6)

Total	899	2.44 (0.00 to 6.69)	273 (30.7)	615 (69.3)	64 (7.2)	306 (34.7)	513 (58.1)	2.72	460 (52.9)	410 (47.1)

^a^Distant disease free survival times in years and (range). ^b^Numbers outside parenthesis represent counts while those inside are percentages of the total number in each outcome group. DDFS, distant disease-free survival; ER, ostrogen receptor; pN, pathological nodal; POSH, Prospective study of Outcomes in Sporadic versus Hereditary breast cancer.

Data on ER status (positive/negative), histological grade (1 to 3), invasive tumour diameter (not including ductal carcinom *in situ *component 0.5 to 180 mm), and axillary nodal status (1/0) were available for 828 (92.1%) of the cases passing the quality control procedure. We were therefore able to determine whether SNPs with significant impacts on survival were independent of the known prognostic factors by treating them as covariates in Cox's proportional hazards model. For each SNP genotype, hazard ratios (HRs) and 95% confidence intervals (CIs) were determined using the common allele homozygotes as the reference group. For SNPs with 10 or fewer minor allele homozygotes, the minor allele homozygotes were combined with heterozygotes. Dominant and recessive disease models were also tested, and the assumption of proportional hazards was tested using log-log plots.

To search for SNPs affecting tumour biology, we stratified the 899 cases into major subgroups on the basis of ER status (positive or negative) and grade (1 or 3). Because tumour grade is necessarily a subjective classification and some grade 2 tumours have gene expression signatures similar to those of grade 1 or grade 3 tumours [24], many grade 2 tumours might be reclassified as either grade 1 or 3. We therefore elected to compare the extremes of grade 1 versus grade 3. The 133 SNPs were individually tested for association using Cochran-Armitage trend test and two-by-three tables of disease-by-genotype.

Although our study was not designed primarily to detect SNPs that increase breast cancer risk, we explored risk associations by contrasting POSH cases with genotyping data in controls that were obtained from the Wellcome Trust Case Control Consortium (WTCCC) [25]. Multidimensional scaling analysis of the WTCCC and HapMap data involving 60,364 SNPs was used to infer Western European ancestry for 2,980 of the 3,000 WTCCC controls from the National Blood Service and the 1958 British Birth Cohorts. The 28 SNPs that were typed in both POSH and WTCCC datasets [see Additional data file 1] were individually tested for association using Cochran-Armitage trend test and two-by-three tables of disease-by-genotype to contrast POSH cases with WTCCC controls.

To determine accurate levels of significance, it is important to correct for the number of tests performed. However, the most effective approach to correct for multiple tests has yet to be defined. The HapMap Consortium has proposed that adjustments should be made for the total number of independent tests. For tests of prognosis and tumour biology, 133 SNPs were used, and 82 of these in 30 genes were determined to be independent by the multidimensional scaling procedure with maximum pair-wise *r*^2 ^values of less than 0.2. For these tests, the threshold for significance is a *P *value of 0.0006 (0.05/82). Only 28 SNPs in 17 genes were tested when searching for SNPs associated with risk for breast cancer. A conservative Bonferroni correction requires *P *values less than 0.003 (0.05/17) to be considered significant for these tests.

We used SPSS 14.0 (SPSS Inc., San Francisco, CA, USA) to perform Kaplan Meier and Cox regressions and PLINK [23] to carry out Cochran-Armitage trend tests and genotypic tests.

Ethics approval for this research was granted by the South West Multi-Centre Research Ethics Committee (MREC/00/6/69). All patients recruited to POSH gave fully informed written consent.

## Results

### Phenotypic characteristics of the POSH cohort

The phenotypic characteristics of this subset of the POSH cohort are based on clinical and pathological assessment performed at the time of diagnosis. The distribution of grade and ER status in 873 cases that passed quality control and have this phenotypic information available are shown in Table 1. Compared with women aged 40 years and older at diagnosis, the subset of POSH study women have nearly 10% more ER-negative tumours (30.9% in POSH versus 21.4% in women aged ≥ 40 years) [26].

### Association of SNPs with breast cancer risk (cases versus controls)

Testing the 28 SNPs that were also typed in the WTCCC confirmed previous associations between risk for breast cancer and the same SNPs in *TOX3 *and *ESR1 *[3,5,27] (Table 2). We also identified an association with rs12693932 in the *CASP8 *gene that has previously been implicated [1]. However, we report an increased risk with rs12693932, whereas the previous study found a protective effect with rs1045485, which failed to pass our quality control procedure. Unfortunately, none of these SNPs remain significant after conservative correction for multiple tests. The SNPs in *FGFR2 *from our study were not typed as part of the WTCCC, and so these could not be included in this analysis.

**Table 2:** Risk of breast cancer comparing 899 POSH cases with 2,980 WTCCC controls

Gene	SNP	Risk allele	Frequency		Trend test		Genotypic test *P *value
					
			Cases	Controls	OR (95% CI)	*P *value	
*TOX3*	rs3803662	A	0.2905	0.2567	1.19 (1.05 to 1.33)	0.0054	0.0188
*CASP8*	rs12693932	T	0.4933	0.4552	1.17 (1.05 to 1.30)	0.0051	0.0144
*ESR1*	rs2228480	A	0.2007	0.2308	0.84 (0.73 to 0.95)	0.0064	0.0053
*ADH1B*	rs1042026	C	0.3068	0.2803	1.13 (1.01 to 1.28)	0.0307	0.0975
*ESR1*	rs3798577	C	0.4983	0.4713	1.11 (1.00 to 1.24)	0.0441	0.1231

CI, confidence interval; OR, odds ratio; POSH, Prospective study of Outcomes in Sporadic versus Hereditary breast cancer; SNP, single nucleotide polymorphism; WTCCC, Wellcome Trust Case Control Consortium.

### SNPs affecting prognosis

**Figure 2:**
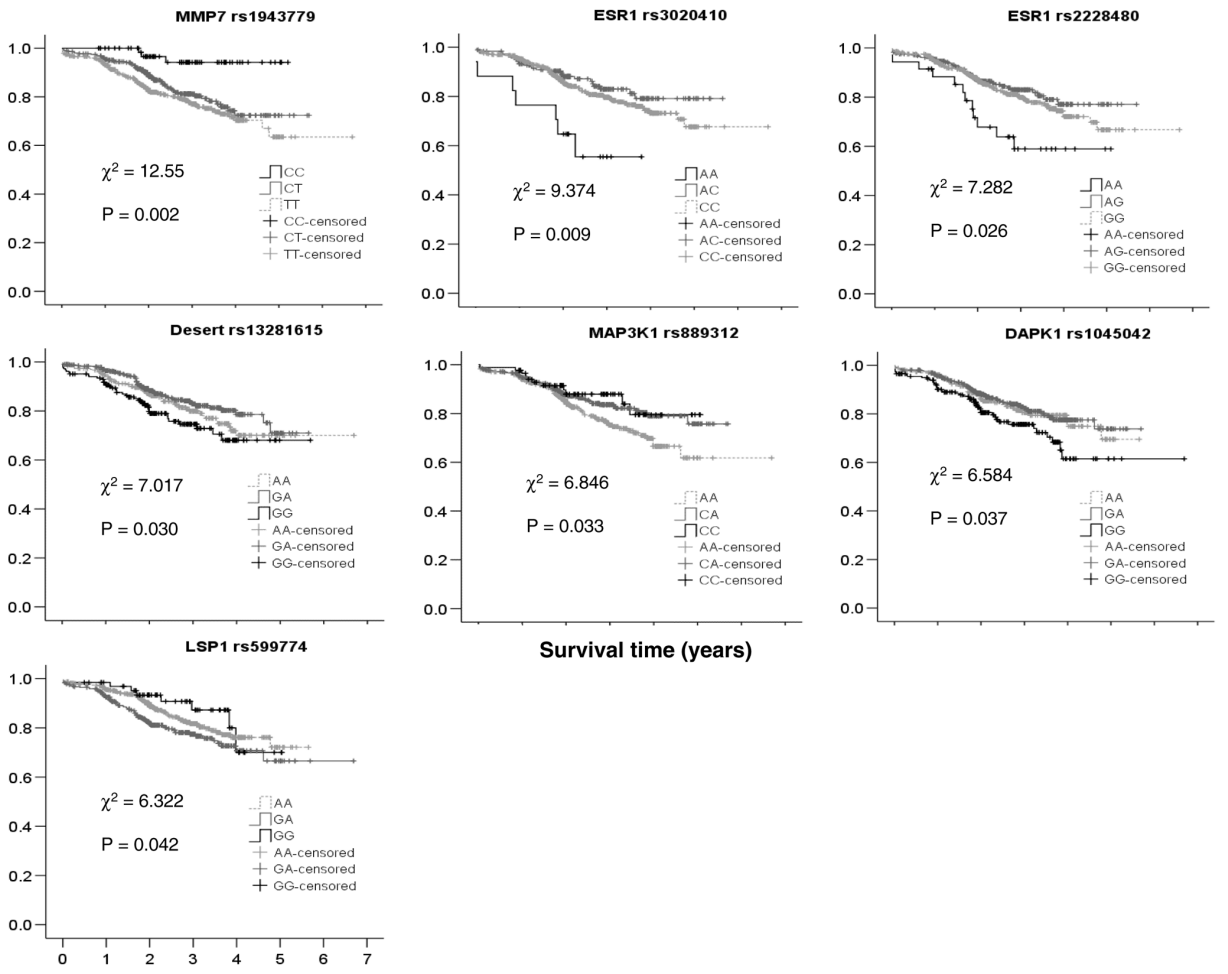
**SNPs affecting prognosis identified by Kaplan-Meier analysis**. Survival curves for rare homozygotes are shown as black lines. The dark grey lines represent survival curves for heterozygotes, and the light grey broken lines depict survival curves for common homozygotes. c^2 ^and associated *P *values are derived from the log-rank test.

To determine whether any of the SNPs affect the duration of DDFS, Kaplan Meier DDFS analysis was conducted in the 133 SNPs that passed the quality control procedure. Before conservative correction for multiple tests, seven of these SNPs, representing six genes, were found to have significant associations with DDFS (Figure 2). Carriers of the minor alleles for five of these SNPs had poorer DDFS, and there was improved DDFS for carriers of two of the SNPs. Data on ER status, histological grade, tumour diameter, and pN stage were available for 828 (92.1%) of the cases passing the quality control procedure. We therefore used Cox regression analysis to determine whether SNPs associated with DDFS were independent of these known prognostic factors by treating them as covariates in the hazard models. After adjustment for ER status, histological grade, tumour diameter and pN stage, three of the seven SNPs identified by the Kaplan Meier analysis remained significant. One additional SNP in *TOX3 *was found to be associated with DDFS by Cox regression (Table 3) but did not reach significance in the Kaplan-Meier analysis. However, these SNPs do not remain significant after conservative correction for the 82 independent tests.

**Table 3:** Summary of most significant SNPs associated with survival independent of known prognostic factors using Cox proportional hazards analysis

Gene/SNP	Genotype	*n*	Events	Censored	Median survival (years)	Hazard ratio	95% CI	*P *value
*MAP3K1*/rs889312	AA	383	76	307	2.393	Reference		
	CA	361	48	313	2.577	0.624	0.435 to 0.897	0.011
	CC	82	10	72	2.508	0.627	0.324 to 1.215	0.166
	Dominant	443	58	385	2.564	0.625	0.443 to 0.881	0.007

*Desert*/rs13281615	AA	280	51	229	2.531	Reference		
	GA	375	49	326	2.517	0.749	0.504 to 1.113	0.153
	GG	170	34	136	2.326	1.349	0.871 to 2.089	0.180
	Recessive	170	34	136	2.326	1.569	1.060 to 2.323	0.024

*MMP7*/rs1943779	TT	429	85	344	2.431	Reference		
	CT	331	47	283	2.477	0.702	0.490 to 1.005	0.053
	CC	67	3	54	2.847	0.217	0.069 to 0.689	0.009
	Dominant	398	50	337	2.539	0.617	0.435 to 0.876	0.007

*TOX3*/rs1420542	AA	551	80	471	2.523	Reference		
	TA	223	47	176	2.487	1.601	1.114 to 2.300	0.011
	TT	41	8	33	1.970	2.069	0.995 to 4.303	0.052
	Dominant	264	55	209	2.407	1.654	1.170 to 2.339	0.004

CI, confidence interval; SNP, single nucleotide polymorphism.

We found a protective effect against the development of distant metastases among cases with the rare C allele of rs1943779 in the *MMP7 *(matrix metalloproteinase 7) gene (rare allele homozygotes HR = 0.217, 95% CI = 0.069 to 0.689; heterozygote HR = 0.702, 95% CI = 0.490 to 1.005) and significant heterogeneity among genotypes (*P *= 0.009).

For the *TOX3 *gene, which has been shown to be associated with an increased risk for breast cancer in published case control studies, the rare allele homozygotes of rs1420542 (TT; n = 41) and heterozygotes (TA; n = 223) showed a 2.1-fold and 1.6-fold risk for distant metastases, respectively, compared with common allele homozygotes. Heterogeneity among genotypes was significant (*P *= 0.013), but this SNP did not achieve statistical significance in the Kaplan-Meier analysis (*P *= 0.077).

The SNP rs889312 in *MAP3K1 *(which encodes MEK kinase) showed a significant improvement in DDFS for heterozygotes of this SNP (HR = 0.624, 95% CI = 0.435 to 0.897) and appears to demonstrate a dominant effect, but DDFS in rare homozygotes (HR = 0.627, 95% CI 0.324 to 1.215) was not significant, despite significant heterogeneity between genotypes (*P *= 0.027).

For rs13281615 at chromosome location 8q24 (no known genes), the evidence for effects that are independent of the known prognostic factors is much weaker because significant results were only obtained for the recessive disease model (HR = 1.569, 95% CI = 1.060 to 2.323).

For the four remaining SNPs that did not show significant associations after correcting for ER, grade, tumour size and pN stage, further Cox regressions were performed that treated these factors separately to determine which of them are correlated with the SNP's effect on DDFS. The survival effects of SNPs in *ESR1 *and *LSP1 *were no longer significant after grade and pN stage were accounted for, whereas the survival effect associated with the *DAPK1 *SNP was attributable to ER status, using Cox regression analysis.

### Association of genotype with tumour phenotype

To confirm the relationships between SNP genotypes and prognostic tumour phenotypes and to search for additional SNPs, we stratified the 899 cases into major subgroups on the basis of ER status (positive or negative) and grade (1 or 3) and tested the 133 SNPs for association using Cochran-Armitage trend test and genotypic tests. Nine SNPs have nominally significant associations, five with ER status and four with grade (Table 4). Of these, three remain significant after Bonferroni correction for the 82 independent SNPs tested (rs2981582 and rs1219648 in *FGFR2*, and rs1801516 in *ATM*, with *P *< 0.0006) and four were in genes shown to have significant effects on the risk for developing distant metastasis (rs1420546 in *TOX3*, rs2228480 and rs3798577 in *ESR1*, and rs661348 in *LSP1*). Comparison of genotypes in patients developing ER-negative tumours with those developing ER-positive tumours demonstrated that SNPs in *FGFR2 *and *TOX3 *are associated with the development of ER-positive breast cancers. In contrast, SNPs in *ATM *were strongly associated with ER-negative breast cancer (Table 4). Comparison of genotypic extremes of grade (grade 1 versus grade 3) demonstrated a significant difference in genotype frequencies for *ESR1*, *IGF1 *and *LSP1 *(Table 4).

**Table 4:** Comparison of extremes of phenotypic subgroups

Phenotype	Gene	SNP	Risk allele	Frequency		Trend test		Genotypic *P *value
						
				ER positive	ER negative	*P *value	OR (95% CI)	
ER (+/-)	*FGFR2*	rs2981582	A	0.478	0.3608	0.000003*	1.62 (1.32 to 2.00)	0.000014
	*FGFR2*	rs1219648	G	0.4861	0.3736	0.000008*	1.59 (1.29 to 1.95)	0.000039
	*ATM*	rs1801516	A	0.1085	0.1776	0.000273*	0.56 (0.42 to 0.75)	0.000402
	*TOX3*	rs1420546	C	0.3655	0.2865	0.002135	1.44 (1.15 to 1.79)	0.003002
	*ATM*	rs3092991	G	0.1248	0.1813	0.002561	0.64 (0.49 to 0.85)	0.00212

Grade (1/3)	*ESR1*	rs2228480	A	0.2178	0.1032	0.002962	2.42 (1.34 to 4.38)	NA
	*ESR1*	rs3798577	C	0.5088	0.373	0.004411	1.74 (1.19 to 2.55)	0.002136
	*IGF1*	rs2373721	G	0.25	0.1406	0.007009	2.04 (1.21 to 3.42)	NA
	*LSP1*	rs661348	C	0.3694	0.4921	0.009682	0.60 (0.42 to 0.88)	0.01541

The genotypic test is not performed if at least one of the cells has a frequency less than 5 which is represented by NA. *Significant SNPs after correcting for 82 independent tests. CI, confidence interval; ER, oestrogen receptor; OR, odds ratio; SNP, single nuceotide polymorphism.

## Discussion

This study utilizes a large group of breast cancer cases derived from the POSH study cohort [20] to identify common variants associated with clinical prognostic tumour characteristics and duration of DDFS. The early age of onset, minimal number of cases carrying high risk genes, and preservation of the natural history of tumours offer a unique opportunity to study young onset breast cancer. Our results from this young onset group of patients are in line with several previous significant findings. For example, SNPs in *TOX3 *and *CASP8 *alter the risk for developing breast cancer, and SNPs in *FGFR2 *and *TOX3 *are strongly associated with ER-positive breast cancer [1,3,10]. These observations remain significant after stringent correction for multiple tests (Table 4) and reinforce the importance of distinguishing between subtypes of breast cancer.

Novel findings presented in this pilot study include the observation that SNP rs1943779 in the *MMP7 *gene is associated with improved DDFS after breast cancer diagnosis. Two adjacent SNPs in the ERα gene affect tumour grade and DDFS. Although these results are significant, they fail to pass correction for multiple testing and require replication in larger studies.

Our observations in the *ESR1 *gene are interesting and may have therapeutic implications. *ESR1 *encodes the ERα, and its expression in tumour cells is a prognostic and predictive factor that is routinely used when making decisions on hormonal therapy. ER-negative tumours have poorer prognosis when compared with ER-positive ones. This is largely due to the higher rate of recurrence in the first 5 years after diagnosis [28]. ER-negative tumours do not respond to oestrogen modulating therapies. Many, although not all, ER-positive tumours respond to medical and surgical approaches that reduce or block the effects of endogenous oestrogen. We observed two SNPs in the 3' region of *ESR1 *that are associated with breast cancer risk, namely rs2228480 and rs3798577. The strength of linkage disequilibrium between these SNPs is low (*r*^2 ^= 0.2), despite them being separated by just over 1 kilobase. The G allele of rs2228480 and the C allele of a different SNP, rs3020410 in the 5' region of *ESR1*, are associated with an increased risk for relapse. Although there was no association of these SNPs with tumour ER status, the difference in survival between genotypes was most significant when examining only patients with ER-positive tumours (for rs2228480, survival analysis for the ER-positive tumours only gave χ^2 ^= 9.12 [*P *= 0.01] and that for the ER-negative tumours yielded χ^2 ^= 0.5 [*P *= 0.78]).

Data on treatment with anti-oestrogenic therapies were available for 99% of the patients genotyped in this study. For those with ER-positive tumours, 93% received adjuvant therapy with anti-oestrogenic modalities, mainly tamoxifen. There are two possible mechanisms through which this survival effect may be mediated. Because there is an association with increased grade, the effect may be through influencing the rate of proliferation of tumours in response to endogenous oestrogen (all patients were premenopausal). Alternatively, the outcome may reflect an effect of this SNP on responsiveness to anti-oestrogen therapies – particularly tamoxifen. Whatever the functionally relevant variant in this region, it is unlikely to be any of the three alleles typed in this study because rs2228480 is a synonymous variant (no change in amino acid coded), rs3798577 is in the 3' untranslated region of the gene, and rs3020410 is intronic.

We also identified four SNPs that may be novel predictors of outcome that are independent of known clinical prognostic factors. The rs1943779 SNP is in the promoter region of *MMP7 *and was found to be associated with improved survival. Proteins of the matrix metalloproteinase family are involved in the breakdown of extracellular matrix in normal physiological processes and tissue remodelling, as well as in disease processes, such as tumour metastasis. It is therefore plausible that variants in matrix metalloproteinase genes may lead to variable responses of the extracellular matrix to tumour invasion – either increasing or decreasing the likelihood of tumour metastasis. More detailed exploration is required to determine whether rs1943779 alters expression of *MMP7*, how this affects tumour invasiveness and whether other closely linked variants are involved.

The protein encoded by *MAP3K1 *(MEK kinase) is a serine/threonine kinase that occupies a pivotal role in a network of phosphorylating enzymes integrating cellular responses to a number of mitogenic and metabolic stimuli. We found that heterozygote carriers of the SNP rs889312 in *MAP3K1 *were more likely to remain free of distant metastases compared with common allele homozygotes, in contrast to a previous study in which heterozygote and minor allele homozygote carriers of this SNP were more likely to have lymph node positive breast cancer at diagnosis [29]. *TOX3 *is an HMG box protein that is involved in chromatin structural modification, and it is one of the low penetrance breast cancer risk genes recently identified in genome-wide association studies [3]. It has also been implicated in breast cancer metastasis to bone [30]. We found that the risk for early relapse with distant metastases was increased in heterozygotes and rare allele homozygotes of SNP rs1420542.

## Conclusion

In this cohort of women with early onset breast cancer, we identified several SNPs in six genes that are associated with the prognostic tumour characteristics of ER status and tumour grade. If the type of breast cancer is determined by background genotype, then this finding has considerable implications for prevention options and also suggests that future association studies should pay careful attention to breast cancer phenotype, because the same risk factors may not be relevant to all types. Furthermore, we have identified three genes associated with the duration of DDFS that are independent of known clinical prognostic factors. Although all of the SNPs tested were from candidate genes and many of these results correspond with previous findings, the results must be treated with caution because only three SNPs associated with ER status remain significant after conservative correction for the number of independent tests performed. Currently, the follow-up time in the POSH cohort is short (median disease-free survival time for disease-free patients is 2.6 years [Table 1]), and so it can only provide an early indication of SNPs that are associated with rapidly progressive disease. Further genotyping in the POSH cohort after longer periods of follow up and among other similar cohorts will determine whether these SNPs remain significant and whether these or different SNPs are associated with more indolent disease progression. Our findings, therefore, need replication in further breast cancer cohorts in which tumour phenotypes and long-term follow-up data are available. If genotyping for low penetrance risk variants is ever to be implemented as a cost-effective public health strategy, it will be important to understand the implications of genetic variants on tumour biology and treatment options as well as for risk across the entire population.

## Abbreviations

CI: confidence interval; DDFS: distant disease-free survival; ER: oestrogen receptor; pN: pathological nodal; HR: hazard ratio; POSH: Prospective study of Outcomes in Sporadic versus Hereditary breast cancer; SNP: single nucleotide polymorphism; WTCCC: Wellcome Trust Case Control Consortium.

## Competing interests

The authors declare that they have no competing interests.

## Authors' contributions

DE is Chief Investigator for the POSH study, conceived the study design and helped to write the manuscript. WT contributed to study design, led the quality control and data analysis, and helped to write the manuscript. VH researched the SNPs to be genotyped, prepared samples and was involved in the data analysis. SG collated the POSH database and extracted relevant phenotypic data. PS gave clinical expertise and helped to revise the manuscript. AC and SE contributed to study design, analysis and helped to write the manuscript.

## Supplementary Material

Additional file 1 An Excel file containing a table listing the number of SNPs genotyped and tested for association with prognosis and risk and the rational for choosing them. Click here for file

## References

[B1] Cox A, Dunning AM, Garcia-Closas M, Balasubramanian S, Reed MW, Pooley KA, Scollen S, Baynes C, Ponder BA, Chanock S, Lissowska J, Brinton L, Peplonska B, Southey MC, Hopper JL, McCredie MR, Giles GG, Fletcher O, Johnson N, Dos SS, Gibson IL, Bojesen SE, Nordestgaard BG, Axelsson CK, Torres D, Hamann U, Justenhoven C, Brauch H, Chang-Claude J, Kropp S (2007). A common coding variant in CASP8 is associated with breast cancer risk. Nat Genet.

[B2] Walsh T, King MC (2007). Ten genes for inherited breast cancer. Cancer Cell.

[B3] Easton DF, Pooley KA, Dunning AM, Pharoah PDP, Thompson D, Ballinger DG, Struewing JP, Morrison J, Field H, Luben R, Wareham N, Ahmed S, Healey CS, Bowman R, Meyer KB, Haiman CA, Kolonel LK, Henderson BE, Le Marchand L, Brennan P, Sangrajrang S, Gaborieau V, Odefrey F, Shen CY, Wu PE, Wang HC, Eccles D, Evans DG, Peto J, Fletcher O (2007). Genome-wide association study identifies novel breast cancer susceptibility loci. Nature.

[B4] Campeau PM, Foulkes WD, Tischkowitz MD (2008). Hereditary breast cancer: new genetic developments, new therapeutic avenues. Hum Genet.

[B5] Stacey SN, Manolescu A, Sulem P, Rafnar T, Gudmundsson J, Gudjonsson SA, Masson G, Jakobsdottir M, Thorlacius S, Helgason A, Aben KK, Strobbe LJ, bers-Akkers MT, Swinkels DW, Henderson BE, Kolonel LN, Le ML, Millastre E, Andres R, Godino J, Garcia-Prats MD, Polo E, Tres A, Mouy M, Saemundsdottir J, Backman VM, Gudmundsson L, Kristjansson K, Bergthorsson JT, Kostic J (2007). Common variants on chromosomes 2q35 and 16q12 confer susceptibility to estrogen receptor-positive breast cancer. Nat Genet.

[B6] Lakhani SR, Reis-Filho JS, Fulford L, Penault-Llorca F, Vijver M van der, Parry S, Bishop T, Benitez J, Rivas C, Bignon YJ, Chang-Claude J, Hamann U, Cornelisse CJ, Devilee P, Beckmann MW, Nestle-Krämling C, Daly PA, Haites N, Varley J, Lalloo F, Evans G, Maugard C, Meijers-Heijboer H, Klijn JG, Olah E, Gusterson BA, Pilotti S, Radice P, Scherneck S, Sobol H (2005). Prediction of BRCA1 status in patients with breast cancer using estrogen receptor and basal phenotype. Clin Cancer Res.

[B7] Jonsson G, Naylor TL, Vallon-Christersson J, Staaf J, Huang J, Ward MR, Greshock JD, Luts L, Olsson H, Rahman N, Stratton M, Ringner M, Borg A, Weber BL (2005). Distinct genomic profiles in hereditary breast tumors identified by array-based comparative genomic hybridization. Cancer Res.

[B8] Wessels LF, van WT, Hart AA, van't Veer LJ, Reinders MJ, Nederlof PM (2002). Molecular classification of breast carcinomas by comparative genomic hybridization: a specific somatic genetic profile for BRCA1 tumors. Cancer Res.

[B9] Lakhani SR (1999). The pathology of familial breast cancer: morphological aspects. Breast Cancer Res.

[B10] Garcia-Closas M, Hall P, Nevanlinna H, Pooley K, Morrison J, Richesson DA, Bojesen SE, Nordestgaard BG, Axelsson CK, Arias JI, Milne RL, Ribas G, Gonzalez-Neira A, Benitez J, Zamora P, Brauch H, Justenhoven C, Hamann U, Ko YD, Bruening T, Haas S, Dork T, Schurmann P, Hillemanns P, Bogdanova N, Bremer M, Karstens JH, Fagerholm R, Aaltonen K, Aittomaki K (2008). Heterogeneity of breast cancer associations with five susceptibility loci by clinical and pathological characteristics. PLoS Genet.

[B11] Antoniou AC, Spurdle AB, Sinilnikova OM, Healey S, Pooley KA, Schmutzler RK, Versmold B, Engel C, Meindl A, Arnold N, Hofmann W, Sutter C, Niederacher D, Deissler H, Caldes T, Kampjarvi K, Nevanlinna H, Simard J, Beesley J, Chen X, Neuhausen LS, Rebbeck TR, Wagner T, Lynch HT, Isaacs C, Weitzel J, Ganz PA, Daly MB, Tomlinson G, Olopade OI (2008). Common breast cancer-predisposition alleles are associated with breast cancer risk in BRCA1 and BRCA2 mutation carriers. Am J Hum Genet.

[B12] Gudmundsson J, Sulem P, Manolescu A, Amundadottir LT, Gudbjartsson D, Helgason A, Rafnar T, Bergthorsson JT, Agnarsson BA, Baker A, Sigurdsson A, Benediktsdottir KR, Jakobsdottir M, Xu J, Blondal T, Kostic J, Sun J, Ghosh S, Stacey SN, Mouy M, Saemundsdottir J, Backman VM, Kristjansson K, Tres A, Partin AW, bers-Akkers MT, Godino-Ivan Marcos J, Walsh PC, Swinkels DW, Navarrete S, Isaacs SD (2007). Genome-wide association study identifies a second prostate cancer susceptibility variant at 8q24. Nat Genet.

[B13] Carey LA, Perou CM, Livasy CA, Dressler LG, Cowan D, Conway K, Karaca G, Troester MA, Tse CK, Edmiston S, Deming SL, Geradts J, Cheang MC, Nielsen TO, Moorman PG, Earp HS, Millikan RC (2006). Race, breast cancer subtypes, and survival in the Carolina Breast Cancer Study. JAMA.

[B14] Bowen RL, Duffy SW, Ryan DA, Hart IR, Jones JL (2008). Early onset of breast cancer in a group of British black women. Br J Cancer.

[B15] Hartman M, Lindstrom L, Dickman PW, Adami HO, Hall P, Czene K (2007). Is breast cancer prognosis inherited?. Breast Cancer Res.

[B16] Fagerholm R, Hofstetter B, Tommiska J, Aaltonen K, Vrtel R, Syrjakoski K, Kallioniemi A, Kilpivaara O, Mannermaa A, Kosma VM, Uusitupa M, Eskelinen M, Kataja V, Aittomaki K, von SK, Heikkila P, Lukas J, Holli K, Bartkova J, Blomqvist C, Bartek J, Nevanlinna H (2008). NAD(P)H:quinone oxidoreductase 1 NQO1*2 genotype (P187S) is a strong prognostic and predictive factor in breast cancer. Nat Genet.

[B17] Schroth W, Antoniadou L, Fritz P, Schwab M, Muerdter T, Zanger UM, Simon W, Eichelbaum M, Brauch H (2007). Breast cancer treatment outcome with adjuvant tamoxifen relative to patient CYP2D6 and CYP2C19 genotypes. J Clin Oncol.

[B18] Cuzick J, Forbes JF, Sestak I, Cawthorn S, Hamed H, Holli K, Howell A (2007). Long-term results of tamoxifen prophylaxis for breast cancer: 96-month follow-up of the randomized IBIS-I trial. J Natl Cancer Inst.

[B19] Anders CK, Hsu DS, Broadwater G, Acharya CR, Foekens JA, Zhang Y, Wang Y, Marcom PK, Marks JR, Febbo PG, Nevins JR, Potti A, Blackwell KL (2008). Young age at diagnosis correlates with worse prognosis and defines a subset of breast cancers with shared patterns of gene expression. J Clin Oncol.

[B20] Eccles D, Gerty S, Simmonds P, Hammond V, Ennis S, Altman DG, Steering Group (2007). Prospective study of Outcomes in Sporadic versus Hereditary breast cancer (POSH): Study Protocol. BMC Cancer.

[B21] Williams C, Brunskill S, Altman D, Briggs A, Campbell H, Clarke M, Glanville J, Gray A, Harris A, Johnston K, Lodge M (2006). Cost-effectiveness of using prognostic information to select women with breast cancer for adjuvant systemic therapy. Health Technol Assess.

[B22] International HapMap Consortium (2005). A haplotype map of the human genome. Nature.

[B23] Purcell S, Neale B, Todd-Brown K, Thomas L, Ferreira MA, Bender D, Maller J, Sklar P, de Bakker PI, Daly MJ, Sham PC (2007). PLINK: a tool set for whole-genome association and population-based linkage analyses. Am J Hum Genet.

[B24] Ivshina AV, George J, Senko O, Mow B, Putti TC, Smeds J, Lindahl T, Pawitan Y, Hall P, Nordgren H, Wong JE, Liu ET, Bergh J, Kuznetsov VA, Miller LD (2006). Genetic reclassification of histologic grade delineates new clinical subtypes of breast cancer. Cancer Res.

[B25] Wellcome Trust Case Control Consortium (2007). Genome-wide association study of 14,000 cases of seven common diseases and 3,000 shared controls. Nature.

[B26] Li CI, Daling JR, Malone KE (2003). Incidence of invasive breast cancer by hormone receptor status from 1992 to 1998. J Clin Oncol.

[B27] Gold B, Kalush F, Bergeron J, Scott K, Mitra N, Wilson K, Ellis N, Huang H, Chen M, Lippert R, Halldorsson BV, Woodworth B, White T, Clark AG, Parl FF, Broder S, Dean M, Offit K (2004). Estrogen receptor genotypes and haplotypes associated with breast cancer risk. Cancer Res.

[B28] Anderson WF, Chen BE, Jatoi I, Rosenberg PS (2006). Effects of estrogen receptor expression and histopathology on annual hazard rates of death from breast cancer. Breast Cancer Res Treat.

[B29] Huijts PE, Vreeswijk MP, Kroeze-Jansema KH, Jacobi CE, Seynaeve C, Krol-Warmerdam EM, Wijers-Koster PM, Blom JC, Pooley KA, Klijn JG, Tollenaar RA, Devilee P, van Asperen CJ (2007). Clinical correlates of low-risk variants in FGFR2, TNRC9, MAP3K1, LSP1 and 8q24 in a Dutch cohort of incident breast cancer cases. Breast Cancer Res.

[B30] Smid M, Wang Y, Klijn JG, Sieuwerts AM, Zhang Y, Atkins D, Martens JW, Foekens JA (2006). Genes associated with breast cancer metastatic to bone. J Clin Oncol.

[B31] POSH Steering group. http://www.som.soton.ac.uk/research/cancersciences/Groups/molecular_and_genetics/POSH/.

